# The Administration of Panax Ginseng Berry Extract Attenuates High-Fat-Diet-Induced Sarcopenic Obesity in C57BL/6 Mice

**DOI:** 10.3390/nu14091747

**Published:** 2022-04-22

**Authors:** Ji-Eun Shin, So-Hyun Jeon, Sang-Jun Lee, Se-Young Choung

**Affiliations:** 1 Department of Life and Nanopharmaceutical Sciences, Graduate School, Kyung Hee University, Seoul 02447, Korea; cindy@khu.ac.kr; 2 Department of Biomedical and Pharmaceutical Sciences, Graduate School, Kyung Hee University, 26, Kyungheedae-ro, Dongdaemun-gu, Seoul 02447, Korea; yrs02223@daum.net; 3 Holistic Bio Co., Seongnam 13494, Korea; leesjun@holistic-bio.com; 4 Department of Preventive Pharmacy and Toxicology, College of Pharmacy, Kyung Hee University, 26, Kyungheedae-ro, Dongdaemun-gu, Seoul 02447, Korea

**Keywords:** ginseng berry extract, sarcopenic obesity, PI3K/Akt pathway, protein synthesis, degradation, inflammation

## Abstract

Sarcopenia and obesity are serious health problems that are highly related to several metabolic diseases. Sarcopenic obesity, a combined state of sarcopenia and obesity, results in higher risks of metabolic diseases and even mortality than sarcopenia or obesity alone. Therefore, the development of therapeutic agents for sarcopenic obesity is crucial. C57BL/6 mice were fed with a high-fat diet (HFD) for 9 weeks. Then, mice were administered with Panax ginseng berry extract (GBE) for an additional 4 weeks, with continuous HFD intake. GBE significantly decreased the food efficiency ratio, serum lipid and insulin levels, adipose tissue weights, and adipocyte size. It significantly increased the grip strength, muscle masses, and myofiber cross-sectional area. It deactivated the protein kinase C (PKC) theta and zeta, resulting in activation of the phosphoinositide 3-kinase (PI3K)/protein kinase B (Akt) pathway, which is known to regulate muscle synthesis and degradation. Furthermore, it inhibited the production of inflammatory cytokines in the muscle tissue. GBE attenuated both obesity and sarcopenia. Thus, GBE is a potential agent to prevent or treat sarcopenic obesity.

## 1. Introduction

Sarcopenia, a decline in skeletal muscle mass and strength, is an important health problem, prevalent in elderly people [1]. Obesity is also a crucial health concern worldwide, and its prevalence has been continuously increasing [2]. Both obesity and sarcopenia are highly associated with metabolic disorders, disability, and even mortality [3]. Sarcopenic obesity, the combined state of sarcopenia and obesity, leads to higher risks of metabolic diseases, disability, and mortality rates than either sarcopenia or obesity alone [4]. However, the research on the molecular mechanism and therapeutic strategies of sarcopenic obesity are still lacking [5]. Therefore, the development of therapeutics or preventive medicine for sarcopenic obesity is necessary.

The molecular mechanism of sarcopenic obesity is not well understood. In this study, we investigated how the obese state induces sarcopenia. The increased inflow of fatty acid due to the obese state accumulates intramyocellular lipids in muscle tissue [6,7]. These lipids consist of diacylglycerol (DAG), ceramides, triacylglycerol, etc. Increased DAG content activates protein kinase C theta (PKCθ), predominantly expressed in skeletal muscle [8,9]. PKCθ induces serine phosphorylation of insulin receptor substrate-1 (IRS1), resulting in the impairment of insulin signaling. Increased ceramide content activates protein kinase C zeta (PKCζ) and deactivates protein kinase B (Akt), involved in insulin signaling [10,11]. Among the insulin signaling pathways, the phosphoinositide 3-kinase (PI3K)/Akt pathway is known to regulate muscle protein synthesis and degradation [12,13]. Deactivation of the PI3K/Akt pathway inhibits protein synthesis and promotes protein degradation. It causes an imbalance in muscle mass maintenance and induces muscle loss. Further, the accumulation of intramyocellular lipids induces the secretion of inflammatory cytokines, such as tumor necrosis factor-α (TNF-α), interleukin-6 (IL-6), and IL-1β [14,15]. It induces low-level inflammation in the muscle tissue, resulting in a continuous muscle atrophic state. Taken together, increased fatty acid uptake and intramyocellular lipid accumulation in muscle tissue deactivate the PI3K/Akt pathway and induce low-level inflammation, resulting in muscle loss and, ultimately, sarcopenic obesity.

The root of Panax ginseng has long been used as an herbal medicine in Asian counties. It has various pharmacological effects, such as anticancer, antidiabetic, anti-obesity, and anti-inflammation [16,17,18,19]. Its major active components are ginsenosides [20,21,22], and ginsenosides can be obtained from different parts of the plant. Several studies showed that Panax ginseng berry extract (GBE) has higher ginsenoside contents and a distinctive ginsenoside profile compared to Panax ginseng extract [23,24,25]. Therefore, GBE might have higher pharmacological effects than its root against several diseases, but the studies about GBE are insufficient. 

GBE has shown antidiabetic and anti-obesity effects in several studies [26,27,28,29]. However, the effect of GBE on muscle or sarcopenia has not been reported before. Since insulin resistance is highly associated with the cause of sarcopenic obesity and GBE has been reported to have anti-obesity effects, we thought that GBE might have a therapeutic effect on sarcopenic obesity. Therefore, in this study, we investigated the effect of GBE on sarcopenic obesity and aimed to determine its effect on the muscle that is damaged by the obese state. 

## 2. Materials and Methods

### 2.1. Preparation of Panax Ginseng Berry Extract (GBE) and UHPLC-ESI-MS/MS Analysis of GBE

Freshly harvested 4-year-old Korean ginseng berries (Panax ginseng Meyer) cultivated in the Gangwon-do province of South Korea were used. After fresh ginseng berries were water washed, the seeds were removed, and the remainder (pulp and juice) was collected. The remainder was then extracted with 5-times more water than the amount of the remainder at 80 ± 5 °C for 5 h. The extract was filtered and evaporated under a vacuum at 60 °C and spray dried to obtain standardized GBE powder. The production yield of GBE was about 2.5% and the GBE was standardized to contain 5% of the index component, ginsenoside Re. The concentration of seven major ginsenosides in GBE was analyzed by HPLC. Total ginsenoside concentrations (% *w*/*w*) were 15.19%. Individual ginsenoside concentrations were 0.45%, 0.90%, 1.11%, 0.75%, 6.06%, 1.16%, and 0.53% for the ginsenosides Rb1, Rb2, Rc, Rd, Re, Rg1, and Rg2, respectively (described in Supplementary Material, Figure S1).

### 2.2. Animals and Experimental Design

Five-week-old male C57BL/6 mice were purchased from Raon bio. (Yongin, Korea) and housed in a standard animal facility maintained on 12 h:12 h light-dark cycle at 25 ± 1 °C. Mice freely accessed food and water. The animal experiment protocol was approved by the Institutional Animal Care and Use Committee guidelines of Kyung Hee University and the approval number was KHUASP(SE)-19-252. After acclimation, mice were randomly divided into a normal group (n = 8) which was fed with a low-fat diet containing 10% kcal fat (D12450B, Research Diets Inc., New Brunswick, NJ, USA) and CMC (Carboxymethyl cellulose) or a high-fat diet (HFD) feeding group (n = 32) which was fed with a high-fat diet containing 60% kcal fat (D12492, Research Diets Inc., New Brunswick, NJ, USA). The composition of low- and high-fat diets was shown in Supplementary Table S1. After HFD feeding for 9 weeks, the HFD feeding group was randomly divided into four groups as follows (n = 8);

HFD: mice fed with a high-fat diet and administered CMC;

GBE 50: mice fed with a high-fat diet and administered with GBE (50 mg/kg);

GBE 100: mice fed with a high-fat diet and administered with GBE (100 mg/kg);

GBE 200: mice fed with a high-fat diet and administered with GBE (200 mg/kg);

GBE was dissolved in CMC according to the dose of each administration group. Oral administration was performed for 4 weeks. The body weight, food intake, and water intake were measured twice a week throughout the study period.

### 2.3. Measurement of Grip Strength

Grip strength was measured twice a week during an animal experiment using a grip strength test (Bioseb, Chaville, France). Mice were lifted by the tail and placed on the grid connected with the grip-strength test. After the mice held the grid, we pulled them horizontally until the grip was broken. The maximum force of grip was measured and we used the average of five measurements for analysis. The grip strength was normalized to bodyweight [30].

### 2.4. Biochemical Analysis of Serum Lipid and Insulin Levels

At the end of the experiment, mice were anesthetized with isoflurane gas, and blood samples were collected from the inferior vena cava. The blood samples were incubated at room temperature for 30 min and then centrifuged at 3000 rpm for 15 min at 4 °C to obtain serum. Levels of total cholesterol (TC), high-density lipoprotein cholesterol (HDL-c), and triglyceride (TG) were measured using commercial kits purchased from Asan Diagnostics (Seoul, Korea). The low-density lipoprotein cholesterol (LDL-c) level was calculated using Friedewald formula: TC level—HDL-c level—TG level/5. Serum insulin level was analyzed using a commercial kit purchased from the Morinaga Institute of Biological Science (Yokohama, Japan). The serum lipid and insulin levels were measured following the manufacturer’s instructions.

### 2.5. Histological Analysis of Muscle Cross-Sectional Area (CSA) and Adipocyte Size

After sacrifice, the gastrocnemius and epididymal fat were obtained. They were fixed with 4% paraformaldehyde and sliced into 4 μm-thick paraffin-embedded sections. Then, the sections were stained with hematoxylin and eosin (H&E) for 13 h. Stained sections were visualized using an optical microscope (Olympus, Tokyo, Japan). Ten muscle fibers and adipocyte were measured from each image, and the average value was used for quantification using image J software (64-bit Java 1.8.0_172) (n = 10/mice, six mice were measured in each group).

### 2.6. Quantitative Real Time-PCR (qRT-PCR) Assay

Thirty milligrams of quadriceps were homogenized with a liquid nitrogen. A total RNA was extracted using easy-RED™ (iNtRON, Seongnam, Korea) according to the manufacturer’s protocol. Then cDNA was synthesized from the extracted RNA using a PrimeScript™ 1st strand cDNA Synthesis Kit (TaKaRa, Tokyo, Japan). qRT-PCR was performed using a Step One Plus™ Real-Time PCR System (Applied Biosystems, Foster City, CA, USA) with TB Green™ Premix Ex Taq™ (TaKaRa, Tokyo, Japan). The mRNA levels were normalized to the Gapdh and calculated using the comparative method (2^−ΔΔCt^). 

### 2.7. Western Blot Assay

Fifty milligrams of gastrocnemius was homogenized with liquid nitrogen and lysed using a lysis buffer containing cOmplete™ Protease Inhibitor Cocktail and PhosSTOP™ (Roche Diagnostics, Indianapolis, IN, USA). Then the lysates were centrifuged (13,000 rpm, 15 min, 4 °C), and the supernatants were collected. The protein concentrations were measured using a Pierce™ BCA Protein Assay Kit (Thermo Fisher Scientific, Rockford, IL, USA). The same amount of protein was loaded on a 12% or 15% polyacrylamide gel and was transferred to a polyvinylidene fluoride membrane. The primary antibodies used purchased as follows, and diluted as 1:1000 with BSA in TBST. The primary antibodies used purchased as follows, and diluted as 1:1000 with BSA: Cell Signalling (Danvers, MA, USA) (p-IRS1 (#2385), IRS1 (#2382), p-PKCζ (#2060), PKC (#9368), p-PI3K (#4228), p-Mtor (#2971), mTOR (#2972), p-Akt (#9271), Akt (#9272), p-S6K1 (#9205), S6K1 (#9202), p-4E-BP1 (#2855), 4E-BP1 (#9452), p-FoxO3a (#9465), and FoxO3a (#12829)), Abcam (Cambridge, UK) (PI3K (ab191606)), Santa Cruz Biotechnology (Santa Cruz, CA, USA) (p-PKCθ (sc-271920), PKCθ (sc-1680), Atrogin-1 (sc-166806) and MurF1 (sc-398608)), and GeneTex (Irvine, CA, USA) (β-actin (GT5512)). The next day, it was incubated with a horseradish peroxidase-conjugated secondary antibody (GeneTex, Irvine, CA, USA) for 100 min. The secondary antibodies were diluted from 1:600 to 1: 2000 with 5% skim milk in TBST. Then it was visualized using a LAS3000 luminescent image analyzer (Fuji Film, Tokyo, Japan). The protein expression level was analyzed using the Image J software (National Institute of Health, Bethesda, MD, USA) and normalized to the β-actin.

### 2.8. Statistical Analysis

The results were expressed as the mean ± SD. The Shapiro–Wilk normality test was conducted to verify the normality of the data. The normally distributed data were analyzed using one-way ANOVA, followed by Tukey’s post hoc test. Statistical significance was determined by SPSS version 25 statistical software (Chicago, IL, USA) and it was expressed as follows: # *p* < 0.05, ## *p* < 0.01, and ### *p* < 0.001 compared to the normal group. * *p* < 0.05, ** *p* < 0.01, and *** *p* < 0.001 compared to the HFD group. 

## 3. Results

### 3.1. GBE Administration Significantly Decreased Food Efficiency Ratio and Increased Grip Strength

**Figure 1:**
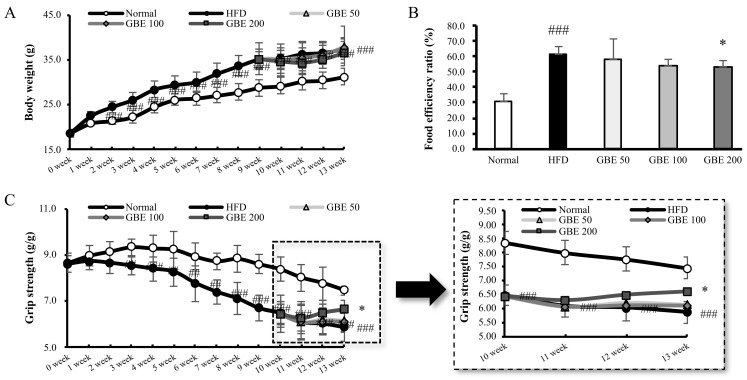
GBE administration significantly decreased food efficiency ratio and increased grip strength. (**A**) The body weight (g) during an animal experiment. (**B**) The food efficiency ratio (FER, %). (**C**) The grip strength (g/g) during an animal experiment. It was expressed as a ratio of grip strength to body weight. Data are expressed as mean ± SD. ## *p* < 0.01 and ### *p* < 0.001 versus the normal group. * *p* < 0.05 versus the HFD group.

At the beginning of the experiment, the body weights of the normal and HFD group were similar. After two weeks of LFD or HFD intake, the body weight in the HFD feeding group was significantly increased compared to the normal group. After nine weeks, the HFD feeding group was divided into HFD, GBE 50, GBE 100, and GBE 200 groups. The body weight did not show significant differences as a result of GBE administration (Figure 1A). To investigate the effect of GBE administration, we measured the food efficiency ratio (FER). FER was calculated using the equation: (The final body weight—the initial body weight (g)/food intake (g) × 100). The initial and final body weight are shown in Supplementary Table S2 and old people and water intake are shown in Supplementary Figure S2. The FER was significantly increased in the HFD group compared to the normal group, and it was significantly decreased in the GBE 200 group compared to the HFD group (Figure 1B). The grip strength was significantly decreased in the HFD feeding group compared to the normal group, after three weeks of HFD intake. At the end of the experiment, the grip strength in the GBE 200 group significantly increased compared to the HFD group (Figure 1C).

### 3.2. GBE Administration Significantly Increased Muscle Mass and Decreased Adipose Tissue Weights

**Figure 2:**
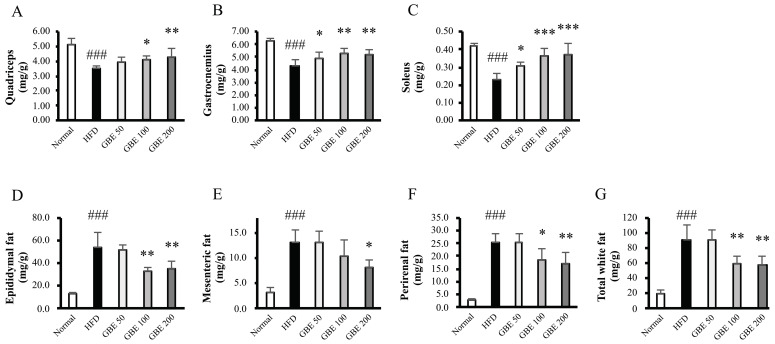
GBE administration significantly increased muscle mass and decreased adipose tissue weights. The muscle mass: (**A**) quadriceps, (**B**) gastrocnemius, and (**C**) soleus. They were expressed as a ratio of muscle tissue weight to body weight (mg/g). The adipose tissue weights: (**D**) epididymal fat, (**E**) mesenteric fat, (**F**) perirenal fat, and (**G**) total white fat. The total white fat was calculated, adding the three fat weights. They were shown as a ratio of fat weight to body weight (mg/g). Data are expressed as mean ± SD. ### *p* < 0.001 versus the normal group. * *p* < 0.05, ** *p* < 0.01, and *** *p* < 0.001 versus the HFD group.

After sacrifice, muscle and adipose tissues were collected and weighed. The muscle tissue weights (quadriceps, gastrocnemius, and soleus) were significantly decreased by HFD feeding and were significantly increased by GBE administration, in a dose-dependent manner (Figure 2A–C). The adipose tissue weights (epididymal, mesenteric, and perirenal fat) were significantly increased by HFD intake and were significantly decreased by GBE treatment, in a dose-dependent manner (Figure 2D–G). 

### 3.3. GBE Administration Significantly Decreased Serum Lipid and Insulin Levels

The serum TC level was significantly increased in the HFD group compared to the normal group and significantly decreased in the GBE 200 group compared to the HFD group. The HDL-c level in the serum showed no significant differences between groups. However, the HDL-c/TC ratio was significantly decreased in the HFD group compared to the normal group, and it was significantly increased by GBE administration, in a dose-dependent manner. The LDL-c level was significantly increased in the HFD group compared to the normal group and was significantly decreased in the GBE 200 group compared to the HFD group. The serum TG level was significantly increased in the HFD group compared to the normal group and significantly decreased in the GBE 200 group compared to the HFD group. The blood insulin level was significantly increased in the HFD group compared to the normal group and was significantly decreased in the GBE 200 group compared to the HFD group (Table 1). 

### 3.4. GBE Administration Significantly Decreased Adipocyte Size and Significantly Increased Myofiber Cross-Sectional Area (CSA)

**Figure 3:**
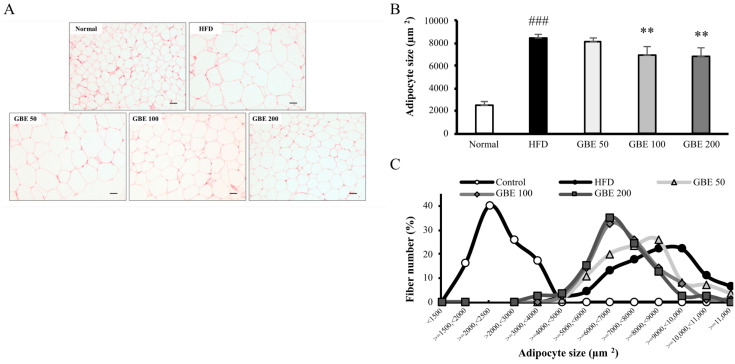
GBE administration significantly decreased adipocyte size. (**A**) H&E staining of epididymal fat. (**B**) The mean adipocyte size of epididymal fat. (**C**) The distribution graph of adipocyte size. Data are expressed as mean ± SD. ### *p* < 0.001 versus the normal group. ** *p* < 0.01 versus the HFD group.

**Figure 4:**
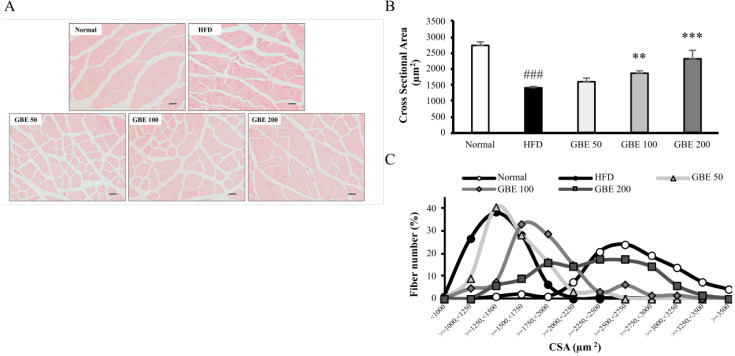
GBE administration significantly decreased significantly increased myofiber cross-sectional area (CSA). (**A**) H&E staining of gastrocnemius. (**B**) The mean adipocyte size of epididymal fat. (**C**) The distribution graph of adipocyte size. Data are expressed as mean ± SD. ### *p* < 0.001 versus the normal group. ** *p* < 0.01 and *** *p* < 0.001 versus the HFD group.

The adipocyte size of epididymal fat and myofiber CSA of gastrocnemius were measured using captured images of H&E-stained sections. The mean adipocyte size was significantly increased in the HFD group compared to the normal group. It was significantly decreased in the GBE 100 and GBE 200 group compared to the HFD group (Figure 3A,B). The adipocyte size distribution graph showed an increase in adipocyte size from HFD feeding and a decrease from GBE treatment (Figure 3C). The average CSA was significantly lower in the HFD group than the normal group. It was significantly increased in the GBE 100 and GBE 200 group compared to the HFD group (Figure 4A,B). The CSA distribution of each myofiber in the HFD group was decreased compared to the normal group. The GBE treatment enlarged it in a dose-dependent manner (Figure 4C).

### 3.5. GBE Administration Up-Regulated the PI3K/Akt Pathway through Inactivation of PKCθ and PKCζ

**Figure 5:**
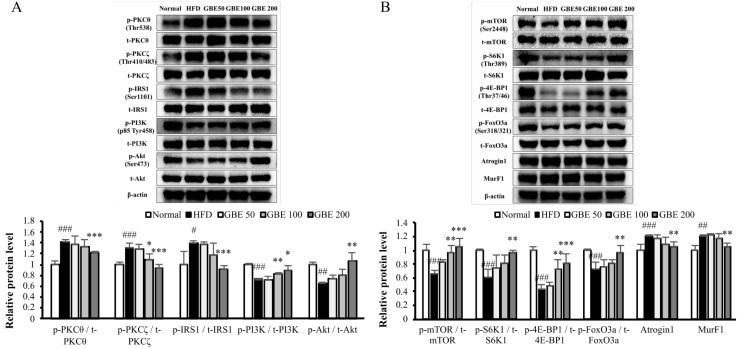
GBE administration up-regulated the PI3K/Akt pathway through inactivation of PKCθ and PKCζ. (**A**) The changes in upstream factors of PI3K/Akt pathway. (**B**) The changes in downstream factors of PI3K/Akt pathway. The protein expression levels were normalized to the β-actin level. Data are expressed as mean ± SD. # *p* < 0.05, ## *p* < 0.01 and ### *p* < 0.001 versus the normal group. * *p* < 0.05, ** *p* < 0.01, and *** *p* < 0.001 versus the HFD group.

Increased lipid accumulation in muscle tissue due to HFD intake activates PKCθ and PKCζ. PKCθ and PKCζ down-regulate the PI3K/Akt pathway, which is known to regulate protein synthesis and degradation. The phosphorylation ratios of PKCθ, PKCζ, and IRS1 were significantly decreased in the HFD group compared to the normal group and significantly increased in the GBE 200 group compared to the HFD group. The phosphorylation ratios of PI3K and Akt were significantly decreased by HFD feeding and significantly increased by GBE 200mg/kg administration (Figure 5A). The activation of PI3K and Akt lead to significant increases in the phosphorylation ratios of the mammalian target of rapamycin (mTOR), ribosomal protein S6 kinase beta-1 (S6K1), and eukaryotic translation initiation factor 4E-binding protein 1 (4E-BP1), which are known to activate protein synthesis. The phosphorylation ratio of forkhead box protein O3 (FoxO3a) was also significantly increased by GBE 200mg/kg administration, resulting in significant decreases in muscle atrophy F-box protein (Atrogin1) and muscle ring finger-1 (MuRF1) expression levels, known to induce protein degradation (Figure 5B).

### 3.6. GBE Administration Down-Regulated the Expression Levels of Inflammatory Cytokines

**Figure 6:**
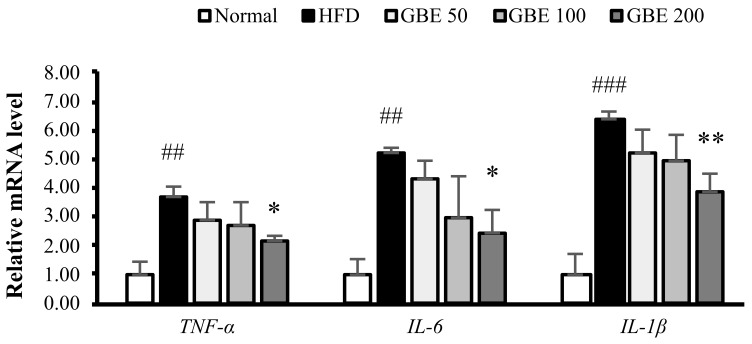
GBE administration down-regulated the expression levels of inflammatory cytokines. The mRNA expression levels were normalized to the gapdh level. Data are expressed as mean ± SD. ## *p* < 0.01, and ### *p* < 0.001 versus the normal group. * *p* < 0.05, ** *p* < 0.01, versus the HFD group.

To investigate the effect of GBE administration on inflammation, we measured the inflammatory cytokine expression levels. TNF-α, IL-6, and IL-1β mRNA levels were significantly increased by HFD feeding and decreased by GBE 200mg/kg administration (Figure 6).

## 4. Discussion

Continuous HFD intake significantly increases body weight, adipose tissue weights, and adipose sizes [31,32], and decreases grip strength, muscle mass, and myofiber CSA [33,34]. In our study, those changes were similarly shown; thus, we confirmed the onset of sarcopenic obesity due to HFD consumption. 

GBE showed an attenuating effect on both sarcopenia and obesity. GBE administration significantly increased the grip strength, muscle mass, and myofiber CSA. It also significantly decreased the serum lipid levels, adipose tissue weights, and adipocyte size. Interestingly, the body weight showed no significant changes as a result of GBE administration. Considering changes in muscle mass and fat weight, it seems that muscle mass increased as the amount of fat decreased. 

GBE was previously studied for its anti-diabetic effect [26,27,28]. It lowered the serum insulin levels and insulin resistance scores (HOMA-IR). When we measured the blood insulin level in our study, it was significantly decreased by GBE administration. Among insulin signaling, the PI3K/Akt pathway is highly associated with the cause of sarcopenia [13,35]. In previous studies, ginsenoside Re reversed insulin resistance through regulating IRS1 and IRS1-bound PI3K [36], and ginsenoside Rg1 attenuated starvation-induced muscle degradation through regulating the Akt/FoxO signaling pathway [37]. Since ginsenoside Re and Rg1 are the major components of GBE, we thought that GBE might affect the PI3K/Akt pathway. Therefore, we investigated the changes in the PI3K/Akt pathway and its association with obesity. The continuous intake of a 6% HFD caused lipid accumulation in muscle tissue and it activated PKCθ and PKCζ [6]. Activated PKCθ and PKCζ deactivates IRS1 and Akt, respectively [7], resulting in deactivation of the PI3K/Akt pathway, which regulates protein synthesis and degradation [38]. GBE administration inactivated PKCθ and PKCζ and, thus, activated IRS1, PI3K, and Akt. When PI3K activates Akt, Akt phosphorylates mTOR and FoxO3a [39,40]. Activated mTOR phosphorylates S6K1 and 4E-BP1, which induce protein synthesis by promoting ribosomal protein S6 and releasing the translation initiation factor eIF4E. GBE administration increased the phosphorylation ratios of mTOR, S6K1, and 4E-BP1. FoxO3a, a transcription factor of two E3 ubiquitin ligases (Atrogin1 and MurF1), is deactivated by phosphorylation [41]. Atrogin1 and MurF1 promote protein degradation through the ubiquitin-proteasome system. Further, increased adipose tissue surrounding the muscle enhances FoxO and up-regulates Atrogin1 and MurF1 [42]. GBE administration deactivated FoxO3a and, thus, decreased Atrogin1 and MurF1 expression levels. In summary, GBE attenuated sarcopenic obesity through deactivation of PKCθ and PKCζ and activation of the PI3K/Akt pathway. It promoted protein synthesis by activating mTOR and inhibited protein degradation by deactivating FoxO3a.

Obesity is a state of chronic inflammation with increased inflammatory cytokine levels in the blood [43]. It affects the muscle tissue and increases the production of inflammatory cytokines in the muscle tissue [44]. When we measured the inflammatory cytokine levels in muscle tissue, HFD intake significantly increased expression levels but GBE administration dose-dependently decreased expression levels. Therefore, GBE administration attenuated sarcopenic obesity by reducing inflammatory cytokine levels. 

## 5. Conclusions

Panax ginseng berry extract attenuates sarcopenic obesity. GBE increased muscle-function-related factors: grip strength, muscle mass, and muscle fiber CSA. It also decreased obesity-related factors: body weight, fat mass, and adipocyte CSA. These results are possible due to two main pathways. First, recovered protein synthesis and degradation imbalance by activating the PI3K/Akt pathway in skeletal muscle. Second, recovered serum lipid and insulin level. In addition, it inhibits the production of inflammatory cytokines in the muscle tissue. Collectively, GBE could be used as an effective natural treatment for sarcopenic obesity.

## Figures and Tables

**Table 1:** Data are expressed as mean ± SD. # *p* < 0.05 ## *p* < 0.01 and ### *p* < 0.001 versus the normal group. * *p* < 0.05 and ** *p* < 0.01 versus the HFD group.

Group		Normal	HFD	GBE 50	GBE 100	GBE 200
Factors						
TC (mg/dL)		105.59 ± 6.74	14.04 ± 14.19 ##	135.20 ± 13.04	125.33 ± 9.87	119.41 ± 11.79 *
HDL-c (mg/dL)		66.74 ± 3.62	80.18 ± 11.06	82.38 ± 9.45	81.28 ± 1.05	79.63 ± 4.65
HDL-c/TC ratio(mg/dL)		0.66 ± 0.03	0.55 ± 0.04 ##	0.61 ± 0.03	0.65 ± 0.04 *	0.67 ± 0.04 **
LDL-c (mg/dL)		29.10 ± 3.80	49.88 ± 11.20 ##	42.37 ± 5.01	34.73 ± 11.01	33.41 ± 7.50 *
TG (mg/dL)		44.00 ± 8.64	71.00 ± 16.77 #	64.00 ± 12.65	47.00 ± 8.87	44.50 ± 8.81 *
Insulin (ng/mL)		0.30 ± 0.03	0.99 ± 0.01 ###	1.09 ± 0.15	0.96 ± 0.04	0.71 ± 0.16 *

## Data Availability

Data is contained within the article and the Supplementary Materials.

## Supplementary Materials

The following are available online at https://www.mdpi.com/article/10.3390/nu14091747/s1, Supplementary Figure S1. UHPLC-ESI-MS/MS profile of GBE, Supplementary Figure S2. (A) The daily water intake and (B) the food intake per animal, Supplementary Table S1. The composition of low- and high-fat diets, Supplementary Table S2. Initial and final body weight.

## References

[1] Kalinkovich A., Livshits G. (2017). Sarcopenic obesity or obese sarcopenia: A cross talk between age-associated adipose tissue and skeletal muscle inflammation as a main mechanism of the pathogenesis. Ageing Res. Rev..

[2] Blüher M. (2019). Obesity: Global epidemiology and pathogenesis. Nat. Rev. Endocrinol..

[3] Wannamethee S.G., Atkins J.L. (2015). Muscle loss and obesity: The health implications of sarcopenia and sarcopenic obesity. Proc. Nutr. Soc..

[4] Choi K.M. (2016). Sarcopenia and sarcopenic obesity. Korean J. Intern. Med..

[5] Zamboni M., Rubele S., Rossi A.P. (2019). Sarcopenia and obesity. Curr. Opin. Clin. Nutr. Metab. Care.

[6] Tumova J., Andel M., Trnka J. (2016). Excess of free fatty acids as a cause of metabolic dysfunction in skeletal muscle. Physiol. Res..

[7] Kitessa S.M., Abeywardena M.Y. (2016). Lipid-induced insulin resistance in skeletal muscle: The chase for the culprit goes from total intramuscular fat to lipid intermediates, and finally to species of lipid intermediates. Nutrients.

[8] Schmitz-Peiffer C., Biden T.J. (2008). Protein kinase C function in muscle, liver, and β-cells and its therapeutic implications for type 2 diabetes. Diabetes.

[9] Morino K., Petersen K.F., Shulman G.I. (2006). Molecular mechanisms of insulin resistance in humans and their potential links with mitochondrial dysfunction. Diabetes.

[10] Lipina C., Hundal H.S. (2017). Lipid modulation of skeletal muscle mass and function. J. Cachexia Sarcopenia Muscle.

[11] Galadari S., Rahman A., Pallichankandy S., Galadari A., Thayyullathil F. (2013). Role of ceramide in diabetes mellitus: Evidence and mechanisms. Lipids Health Dis..

[12] Glass D.J. (2005). Skeletal muscle hypertrophy and atrophy signaling pathways. Int. J. Biochem. Cell Biol..

[13] Vainshtein A., Sandri M. (2020). Signaling pathways that control muscle mass. Int. J. Mol. Sci..

[14] Palomer X., Pizarro-Delgado J., Barroso E., Vázquez-Carrera M. (2018). Palmitic and oleic acid: The yin and yang of fatty acids in type 2 diabetes mellitus. Trends Endocrinol. Metab..

[15] Shou J., Chen P.-J., Xiao W.-H. (2020). Mechanism of increased risk of insulin resistance in aging skeletal muscle. Diabetol. Metab. Syndr..

[16] Helms S. (2004). Cancer prevention and therapeutics: Panax ginseng. Altern. Med. Rev..

[17] Lee H.J., Lee Y.-H., Park S.K., Kang E.S., Kim H.-J., Lee Y.C., Choi C.S., Park S.E., Ahn C.W., Cha B.-S. (2009). Korean red ginseng (*Panax ginseng*) improves insulin sensitivity and attenuates the development of diabetes in Otsuka Long-Evans Tokushima fatty rats. Metabolism.

[18] Kim D.Y., Yang W.M. (2011). Panax ginseng ameliorates airway inflammation in an ovalbumin-sensitized mouse allergic asthma model. J. Ethnopharmacol..

[19] Li Z., Ji G.E. (2018). Ginseng and obesity. J. Ginseng Res..

[20] Sun S., Qi L.-W., Du G.-J., Mehendale S.R., Wang C.-Z., Yuan C.-S. (2011). Red notoginseng: Higher ginsenoside content and stronger anticancer potential than Asian and American ginseng. Food Chem..

[21] Cho W.C., Chung W.-S., Lee S.K., Leung A.W., Cheng C.H., Yue K.K. (2006). Ginsenoside Re of Panax ginseng possesses significant antioxidant and antihyperlipidemic efficacies in streptozotocin-induced diabetic rats. Eur. J. Pharmacol..

[22] Kim J.H., Yi Y.-S., Kim M.-Y., Cho J.Y. (2017). Role of ginsenosides, the main active components of Panax ginseng, in inflammatory responses and diseases. J. Ginseng Res..

[23] Kim C.-K., Cho D.H., Lee K.-S., Lee D.-K., Park C.-W., Kim W.G., Lee S.J., Ha K.-S., Taeg O.G., Kwon Y.-G. (2012). Ginseng berry extract prevents atherogenesis via anti-inflammatory action by upregulating phase II gene expression. Evid.-Based Complementary Altern. Med..

[24] Kim Y.K., Yoo D.S., Xu H., Park N.I., Kim H.H., Choi J.E., Park S.U. (2009). Ginsenoside content of berries and roots of three typical Korean ginseng (*Panax ginseng*) cultivars. Nat. Prod. Commun..

[25] Cho K.S., Park C.W., Kim C.-K., Jeon H.Y., Kim W.G., Lee S.J., Kim Y., Lee J., Choi Y. (2013). Effects of Korean ginseng berry extract (GB0710) on penile erection: Evidence from in vitro and in vivo studies. Asian J. Androl..

[26] Xie J., Zhou Y.-P., Dey L., Attele A., Wu J., Gu M., Polonsky K., Yuan C.-S. (2002). Ginseng berry reduces blood glucose and body weight in db/db mice. Phytomedicine.

[27] Attele A.S., Zhou Y.-P., Xie J.-T., Wu J.A., Zhang L., Dey L., Pugh W., Rue P.A., Polonsky K.S., Yuan C.-S. (2002). Antidiabetic effects of Panax ginseng berry extract and the identification of an effective component. Diabetes.

[28] Seo E., Kim S., Lee S.J., Oh B.-C., Jun H.-S. (2015). Ginseng berry extract supplementation improves age-related decline of insulin signaling in mice. Nutrients.

[29] Chae H.-S., You B.H., Choi J., Chin Y.-W., Kim H., Choi H.S., Choi Y.H. (2019). Ginseng berry extract enhances metformin efficacy against obesity and hepatic steatosis in mice fed high-fat diet through increase of metformin uptake in liver. J. Funct. Foods.

[30] Witteveen E., Hoogland I.C., Wieske L., Weber N.C., Verhamme C., Schultz M.J., Van Schaik I.N., Horn J. (2016). Assessment of intensive care unit-acquired weakness in young and old mice: An *E. coli* septic peritonitis model. Muscle Nerve.

[31] Lee M.R., Kim J.E., Choi J.Y., Park J.J., Kim H.R., Song B.R., Choi Y.W., Kim K.M., Song H., Hwang D.Y. (2019). Anti-obesity effect in high-fat-diet-induced obese C57BL/6 mice: Study of a novel extract from mulberry (Morus alba) leaves fermented with Cordyceps militaris. Exp. Ther. Med..

[32] Peng Y., Sun Q., Xu W., He Y., Jin W., Yuan L., Gao R. (2019). Vitexin ameliorates high fat diet-induced obesity in male C57BL/6J mice via the AMPKα-mediated pathway. Food Funct..

[33] Tong T., Kim M., Park T. (2019). α-Ionone attenuates high-fat diet-induced skeletal muscle wasting in mice via activation of cAMP signaling. Food Funct..

[34] Yoo A., Jang Y.J., Ahn J., Jung C.H., Seo H.D., Ha T.Y. (2020). Chrysanthemi Zawadskii var. Latilobum Attenuates Obesity-Induced Skeletal Muscle Atrophy via Regulation of PRMTs in Skeletal Muscle of Mice. Int. J. Mol. Sci..

[35] Gheibi S., Kashfi K., Ghasemi A. (2017). A practical guide for induction of type-2 diabetes in rat: Incorporating a high-fat diet and streptozotocin. Biomed. Pharmacother..

[36] Han D.-H., Kim S.H., Higashida K., Jung S.-R., Polonsky K.S., Klein S., Holloszy J.O. (2012). Ginsenoside Re rapidly reverses insulin resistance in muscles of high-fat diet fed rats. Metabolism.

[37] Li F., Li X., Peng X., Sun L., Jia S., Wang P., Ma S., Zhao H., Yu Q., Huo H. (2017). Ginsenoside Rg1 prevents starvation-induced muscle protein degradation via regulation of AKT/mTOR/FoxO signaling in C2C12 myotubes. Exp. Ther. Med..

[38] Kandarian S.C., Jackman R.W. (2006). Intracellular signaling during skeletal muscle atrophy. Muscle Nerve.

[39] Argadine H.M., Mantilla C.B., Zhan W.-Z., Sieck G.C. (2011). Intracellular signaling pathways regulating net protein balance following diaphragm muscle denervation. Am. J. Physiol.-Cell Physiol..

[40] Schiaffino S., Mammucari C. (2011). Regulation of skeletal muscle growth by the IGF1-Akt/PKB pathway: Insights from genetic models. Skelet. Muscle.

[41] Kang S.-H., Lee H.-A., Kim M., Lee E., Sohn U.D., Kim I. (2017). Forkhead box O3 plays a role in skeletal muscle atrophy through expression of E3 ubiquitin ligases MuRF-1 and atrogin-1 in Cushing’s syndrome. Am. J. Physiol.-Endocrinol. Metab..

[42] Zhu S., Tian Z., Torigoe D., Zhao J., Xie P., Sugizaki T., Sato M., Horiguchi H., Terada K., Kadomatsu T. (2019). Aging-and obesity-related peri-muscular adipose tissue accelerates muscle atrophy. PLoS ONE.

[43] Gregor M.F., Hotamisligil G.S. (2011). Inflammatory mechanisms in obesity. Annu. Rev. Immunol..

[44] Pellegrinelli V., Rouault C., Rodriguez-Cuenca S., Albert V., Edom-Vovard F., Vidal-Puig A., Clément K., Butler-Browne G.S., Lacasa D. (2015). Human adipocytes induce inflammation and atrophy in muscle cells during obesity. Diabetes.

