# Supplementary material for: The Administration of Panax Ginseng Berry Extract Attenuates High-Fat-Diet-Induced Sarcopenic Obesity in C57BL/6 Mice
Source: Nutrients. 2022 Apr 22;14(9):1747. doi: 10.3390/nu14091747 (PMC9099595; doi:10.3390/nu14091747)
Supplement: Supplementary file 1 [file nutrients-14-01747-s001.zip › nutrients-1625795-supplementary.pdf]

UHPLC-ESI-MS/MS analysis of GBE

Each pulverized GB sample (600 mg) was extracted with 6mL of 70% methanol using a Retsch MM400 mixer mill (Retsch GmbH, Haan, Germany), operated at 30 Hz/s for 10 min. Subsequently, the samples were subjected to sonication in an ultrasonic water bath (Power Sonic 305; Hwashin Technology Co., Seoul, Korea) for 5 min and centrifuged at 17000 rpm at 4C for 15 min. The supernatant was filtered using a 0.2-mm polytetrafluoroethylene filter and concentrated using a speed vacuum concentrator (Modulspin 31; Biotron, Incheon, Korea). The samples collected finally were weighed and reconstituted in 70% methanol. The final concentration of the samples was 50 mg/mL for ultrahigh performance liquid chromatography-electrospray ionization-tandem mass spectrometry (UHPLC-ESI-MS/MS) analysis.

An LTQ XL ion trap mass spectrometer (Thermo Fisher Scientific, San Jose, CA, USA) comprising an electrospray interface coupled with a Dionex UltiMate 3000 RS Pump, RS autosampler, RS column compartment, and RS diode array detector (Dionex Corporation, Sunnyvale, CA, USA) was used in this study. The sample, with an injection volume of 10 mL at a constant flow rate of 0.3 mL/min, was separated on a Thermo Scientific Syncronis C18 UHPLC column (100 mm 2.1 mm [i.d.] 1.7 mm [particle size]). (Thermo Fisher Scientific, San Jose, CA, USA) The gradient mobile phase consisted of solvent A (water + 0.1% formic acid) and solvent B (acetonitrile + 0.1% formic acid). The LC gradient was increased from 10% solvent B to 100% solvent B in 15 min, maintained for 3 min, and then re-equilibrated to the initial condition in 4 min. The photodiode array detector was tuned to a wavelength range of 200-600 nm for metabolite detection, managed by a three-dimensional field. The instrument was operated in a full-scan mode with a mass scan range of 150-1500 m/z. The operating parameters were as follows: capillary temperature was 270°C and sheath gas flow and auxiliary gas flow were 40 and 20 arbitrary units, respectively. The conditions in positive ion (and negative ion) mode for ESI were as follows: capillary voltage of 45 kV (31 kV), source voltage of 5 V (4.5 V), and tube lens voltage of 120 V (60 V).

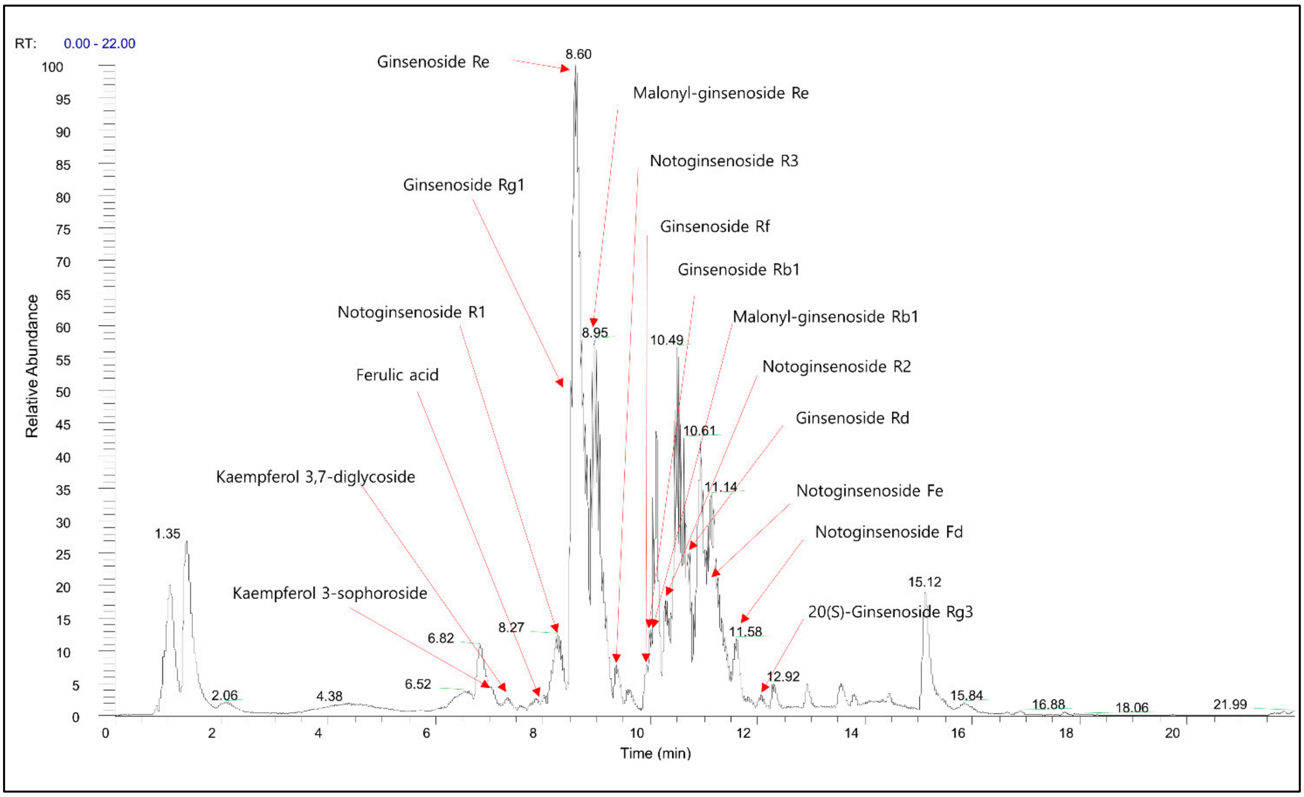

Supplementary Figure S1. UHPLC-ESI-MS/MS profile of GBE

Supplementary Table S1. The composition of low- and high-fat diets

**Formulation**	**Low-fat diet**		**High-fat diet**	
	Ingredients	Grams	Ingredients	Grams
Protein	Casein, Lactic	200g	Casein, Lactic	200g
	Cystine, L	3g	Cystine, L	3g
Carbohydrate	Sucrose	354g	Sucrose	72.8g
	Lodex 10	35g	Lodex 10	125g
	Starch, Corn	315g		
Fiber	Solka Floc, FCC200	50g	Solka Floc, FCC200	50g
Fat	Soybean Oil, USP	25g	Soybean Oil, USP	25g
	Lard	20g	Lard	245g
Mineral•Vitamin•Dye	-	53.05g	-	53.05g
		Total: 1055.05 g		Total: 773.85 g
**Caloric information**	**Low-fat diet**		**High-fat diet**	
Protein	20 % Kcal		20 % Kcal	
Carbohydrate	70 % Kcal		60 % Kcal	
Fat	10 % Kcal		20 % Kcal	
Energy density	3.82 Kcal/g		5.21 Kcal/g	

Supplementary Table S2. Initial and final body weight.

Group / Factors	Normal	HFD	GBE 50	GBE 100	GBE 200
Initial body weight (g)	18.8±0.8	18.7±0.9	18.8±0.7	18.8±1.1	18.7±0.7
Final body weight (g)	31.3±1.8	37.4±2.2	38.1±4.3	37.6±2.3	36.7±2.3

Data are expressed as mean ± SD. #$p < 0.05$ and ## $p < 0.01$ versus to the normal group.

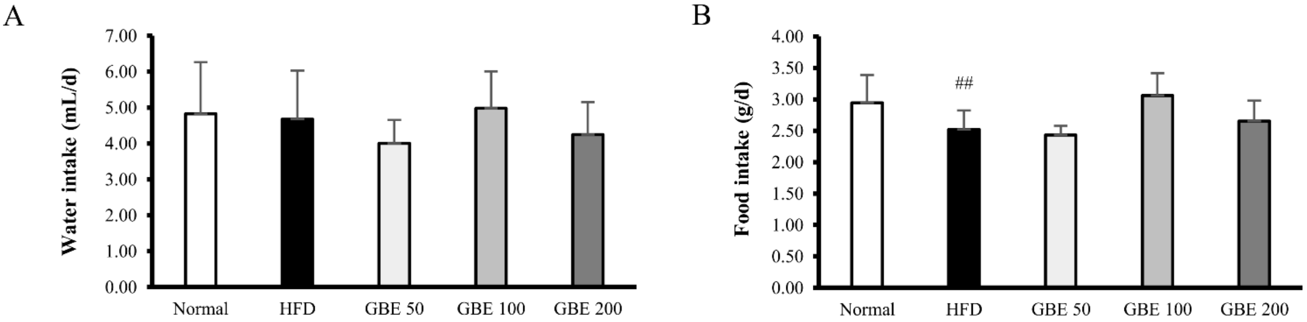

Supplementary Figure S2. (**A**) The daily water intake and (**B**) the food intake per animal.

$p < 0.01$ versus to the normal group.
